# 达雷妥尤单抗治疗晚期轻链型淀粉样变的疗效和安全性

**DOI:** 10.3760/cma.j.issn.0253-2727.2022.01.007

**Published:** 2022-01

**Authors:** 恺妮 沈, 会蕾 苗, 雅娟 高, 欣欣 曹, 道斌 周, 薇 苏, 剑 李

**Affiliations:** 1 中国医学科学院、北京协和医学院北京协和医院血液内科，北京 100730 Department of Hematology, Peking Union Medical College Hospital, Chinese Academy of Medical Sciences and Peking Union Medical College, Beijing 100730, China; 2 中国医学科学院、北京协和医学院北京协和医院检验科，北京 100730 Laboratory Department, Peking Union Medical College Hospital, Chinese Academy of Medical Sciences and Peking Union Medical College, Beijing 100730, China

**Keywords:** 淀粉样变性, 达雷妥尤单抗, 治疗结果, Amyloidosis, Daratumumab, Treatment outcome

## Abstract

**目的:**

回顾性分析晚期轻链（AL）型淀粉样变患者接受达雷妥尤单抗治疗的疗效和安全性。

**方法:**

纳入20例于2017年1月至2021年3月在北京协和医院接受达雷妥尤单抗治疗的初治或复发难治晚期AL型淀粉样变患者。收集患者治疗前基线临床资料及治疗随访资料，分析患者的血液学、器官缓解情况，预后及不良反应。

**结果:**

20例患者的中位年龄为62（45～73）岁，男女比例2.3∶1，9例为初治，11例为复发难治，梅奥2004分期为Ⅱ/Ⅲ期。4例患者于第1个疗程死亡，其余16例患者中位接受3（1～10）个疗程化疗。治疗后1个月、3个月及6个月血液学总缓解率均为80％，≥非常好的部分缓解率分别为45％、60％及60％，中位血液学起效时间为13（6～28）d，3个月、6个月及12个月心脏缓解率分别为20％、30％及40％，中位起效时间为91（30～216）d，1年总生存率为48.4％。至末次随访，9例（45％）患者死亡，1个月内死亡率为25％，Ⅲb期患者1个月内死亡率为40％。3级以上血液学不良反应以淋巴细胞减少最为常见，非血液学不良反应主要为输注反应及感染。

**结论:**

达雷妥尤单抗治疗初治及复发难治晚期AL型淀粉样变可快速诱导高水平的血液学缓解，但Ⅲb期患者仍有较高的早期死亡率。

## Abstract (en)

**Objective:**

The study investigated the efficacy and safety of daratumumab in the treatment of cardiac light chain（AL）amyloidosis.

**Methods:**

We retrospectively analyzed the clinical characteristics, hematologic response, organ response, long-term survival, and adverse events of 20 patients with newly diagnosed or relapsed/refractory cardiac AL amyloidosis treated with daratumumab in Peking Union Medical College Hospitalo from January 2017 to March 2021.

**Results:**

The overall median age of 20 patients was 62（range, 45–73）yeas, with a male to female ratio of 2.3:1. Nine patients were newly diagnosed, while 11 patients had relapsed or refractory disease. Based on Mayo 2004 cardiac AL staging system, stages Ⅱ and Ⅲ diseases were present in 20 patients respectively. Four patients died during the first cycle of daratumumab, and the remaining 16 patients completed a median of 3（range, 1–10）cycles of treatment. Overall hematologic response rates were 80％ each at 1, 3, and 6 months after treatment initiation, and 45％，60％, and 60％ of the patients achieved at least a very good partial response at 1, 3, and 6 months respectively. The median duration to hematologic response was 13（range, 6–28）days. At 3, 6, and 12 months, 20％, 30％, and 40％ of the patients respectively achieved a cardiac response, and the median days to response was 91（range, 30–216）days. As of the last follow-up, 9（45％）patients died. The 1-month mortality rate of all the patients and stage IIIb patients was 25％ and 40％, respectively. The 1-year overall survival rate was 48.4％. Lymphocytopenia was the most common hematological adverse event（above grade 3）. Non-hematological adverse events were mainly infusion-related reactions and infections.

**Conclusion:**

Daratumumab could induce deep and rapid hematologic response in newly diagnosed and previously treated cardiac AL amyloidosis patients. However, daratumumab was not effective in preventing the high and early mortality rate in stage Ⅲb patients.

原发性轻链（AL）型淀粉样变是一种致死性浆细胞病[1]–[2]。近年来，尽管硼替佐米联合化疗显著提高了患者的血液学缓解率及生存率[3]–[4]，但晚期心脏受累患者的早期死亡率仍超过30％[5]–[6]。达雷妥尤单抗是一种人源化抗CD38抗体，国外多项研究已经表明达雷妥尤单抗对于AL型淀粉样变有较好的疗效和安全性[7]–[8]。但国内达雷妥尤单抗上市时间不长，其治疗AL型淀粉样变的经验和数据较少。因此，本研究回顾性分析了我院单中心应用达雷妥尤单抗治疗晚期AL型淀粉样变患者的缓解率及生存情况。

## 病例与方法

1. 病例：纳入2017年1月至2021年3月期间在北京协和医院接受达雷妥尤单抗治疗的初治或复发难治心脏AL型淀粉样变患者，其中复发难治患者指前期接受过至少一线治疗后复发，或从未在前期治疗中达到过部分缓解（PR）及以上的患者，收集患者治疗前的基线临床信息及治疗随访信息。

2. 治疗：所有患者均接受基于达雷妥尤单抗的治疗，达雷妥尤单抗以16 mg/kg或800 mg的固定剂量静脉输注，第1～8周每周1次，第9～16周每2周1次，第17周后每月1次，部分患者由医师根据病情变化调整用药间隔。达雷妥尤单抗输注前予地塞米松、非甾体类抗炎药及抗组胺药预防输注相关反应。Dd方案：达雷妥尤单抗静脉输注联合地塞米松40 mg/d口服（第1、8、15、22天），部分患者单次地塞米松剂量根据心功能减为20 mg。DRd方案：在Dd方案基础上加用来那度胺25 mg/d口服（第1～21天），予阿司匹林100 mg每日1次口服预防血栓。DBd方案：在Dd方案基础上加用硼替佐米1.3 mg/m^2^皮下注射（第1、8、15、22天）。所有患者均接受阿昔洛韦200 mg每日1次口服预防带状疱疹。

3. 疗效及不良反应评估：血液学疗效分为完全缓解（CR）、非常好的部分缓解（VGPR）、PR、血液学稳定（SD）及血液学进展（PD）[9]–[11]。总缓解率（ORR）为CR率、VGPR率及PR率之和。对心脏、肾脏、肝脏受累的患者进行器官疗效评价，分为器官缓解、器官稳定、器官进展[9]。总生存期定义为自接受达雷妥尤单抗治疗至因任何原因死亡的时间。不良反应按照美国常见不良反应术语评定标准5.0版进行评定。

4. 随访：通过查阅患者门诊病例及电话进行随访，随访截止日期为2021年4月30日，中位随访时间9.6（0.1～45.1）个月。

5. 统计学处理：采用SPSS 23.0软件进行统计学分析，连续变量以中位数（范围）的形式描述，分类变量以例数（百分比）的形式描述，生存分析采用Kaplan-Meier法。

## 结果

1. 临床特征及治疗：共纳入20例心脏AL型淀粉样变患者。中位年龄为62（45～73）岁，男女比例2.3∶1，70％的患者为轻链λ型。9例为初治患者，11例为难治复发患者，既往中位治疗线数为1（1～4）线。器官受累方面，肾脏受累占55％，肝脏受累占5％。N末端脑钠肽前体9 935（2 602～35 000）ng/L，24 h尿蛋白0.375（0.060～8.740）g，估算肾小球滤过率66.5（8.0～119.0）ml·min^−1^·（1.73 m^2^）^−1^，碱性磷酸酶92（56～474）U/L，游离轻链差值（dFLC）243.65（86.40～57 486.30）mg/L。各有5例患者为梅奥2004Ⅱ期及Ⅲa期，10例为Ⅲb期。4例接受Dd方案治疗，7例接受DRd方案治疗，其余9例接受DBd方案治疗。4例患者于第1个疗程内死亡，其余16例中位接受3（1～10）个疗程化疗。

2. 疗效：治疗后1个月达CR者1例，达VGPR者8例，达PR者7例，达VGPR及以上患者9例，ORR为80％。治疗后3个月和6个月达CR者2例，达VGPR者10例，达PR者4例，达VGPR及以上患者12例，ORR为80％。随访过程中，dFLC中位降低92.8％，7例（35％）患者实现了治疗后dFLC低于10 mg/L。在获得血液学缓解的患者中，中位起效时间仅为13（6～28）d，达到最佳血液学疗效的中位时间为14（6～215）d（图1）。

**图1:**
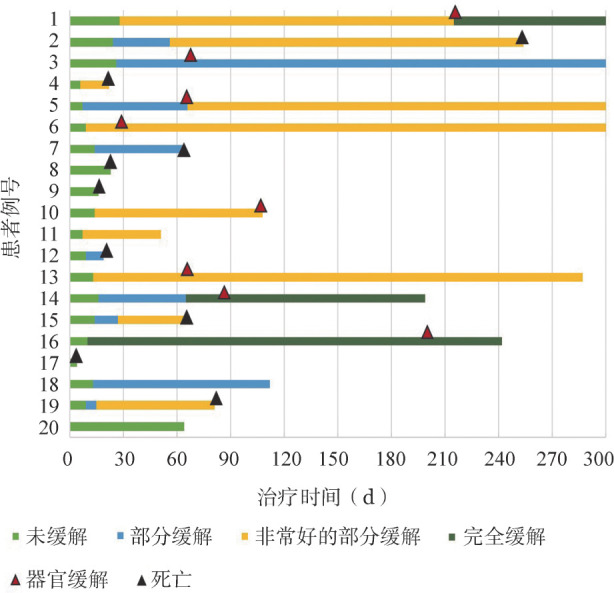
20例晚期轻链型淀粉样变患者的血液学疗效、器官缓解及生存情况

治疗后3个月、6个月及12个月的心脏缓解率分别为20％、30％、40％，达到缓解的中位时间为91（30～216）d。11例肾脏受累的患者中，2例获得器官缓解。1例患者肝脏受累，未获得肝脏缓解。

10例梅奥分期Ⅲb期患者治疗后1个月ORR为70％，其中3例（30％）获得VGPR，4例（40％）获得PR。治疗后3个月1例PR患者的血液学疗效提升至VGPR，治疗后3个月及6个月达VGPR及以上的患者比例为40％。中位血液学起效时间为13（6～24）d，1例患者获得心脏缓解。

3. 生存情况：截至2021年4月30日，9例（45％）患者死亡，患者1个月内死亡率为25％，3个月内死亡率为40％。梅奥Ⅲb期患者1个月内死亡率为40％，3个月内死亡率为50％。所有患者的6个月及12个月总生存率分别为58.0％及48.4％（图2）。

**图2:**
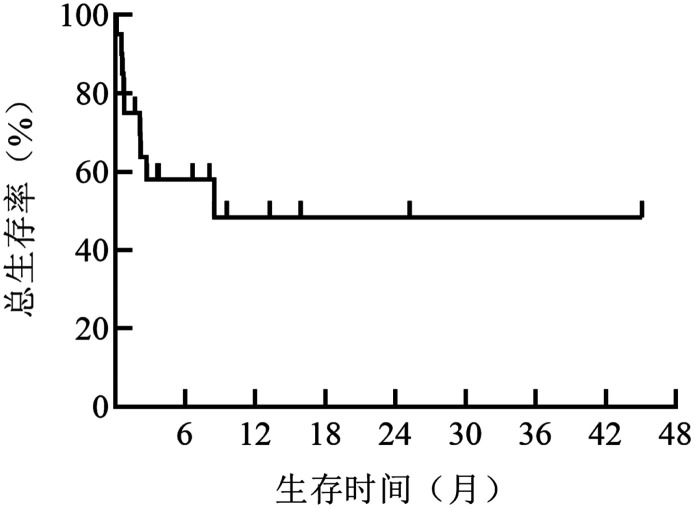
20例晚期轻链型淀粉样变患者的总生存曲线

4. 安全性：3～4级血液学不良反应中，中性粒细胞减少2例（10％），淋巴细胞减少9例（45％），贫血1例（5％）。非血液学不良反应中，6例发生输液相关反应（30％），均为1～2级。感染患者共7例（35％），6例为2级，1例为4级，其中呼吸道感染5例，胃肠道感染2例。

## 讨论

目前治疗AL型淀粉样变的一线化疗方案包括硼替佐米+环磷酰胺+地塞米松和硼替佐米+美法仑+地塞米松等[3]–[4]。高质量血液学缓解率为55％～60％，心脏缓解率约30％，血液学起效的中位时间为1～2个月[5]。心脏受累最严重的Ⅲb期患者早期死亡率仍高于30％，中位生存期仅为3～4个月[12]。我们需要探索更好的治疗方案，以最快的速度诱导最深的血液学缓解，从而改善此类患者的远期生存。

达雷妥尤单抗是一种人源化抗CD38抗原的IgG1κ单克隆抗体，其单药或联合蛋白酶体抑制剂及免疫调节剂已被证实对复发难治多发性骨髓瘤患者有较好的疗效[13]。AL型淀粉样变患者体内的克隆性浆细胞表面同样表达CD38，从而使达雷妥尤单抗治疗AL型淀粉样变患者具备可行性[14]。多项前瞻性研究显示了达雷妥尤单抗治疗复发难治AL型淀粉样变的优势。在一项法国前瞻性Ⅱ期研究中，40例难治复发的Ⅰ～Ⅲa期AL型淀粉样变患者接受了为期24周的达雷妥尤单抗单药治疗，57％患者的血液学疗效达VGPR及以上，心脏缓解率为29％，中位无进展生存期25个月[7]。Sanchorawala等[8]的研究进一步把疗程延长至24个月，血液学疗效达VGPR及以上者占86％，心脏缓解率也提升至50％。德国的一项研究纳入了168例难治复发患者，接受中位5个月的Dd或DVd方案治疗，36％的入组患者为Ⅲb期。结果显示，高质量的血液学缓解率分别为48％及55％，心脏缓解率分别为22％及26％[15]。但达雷妥尤单抗并不能改善Ⅲb期患者的预后。而在初治患者中，ANDROMEDA研究充分阐释了达雷妥尤单抗的优势，不过该研究并没有纳入Ⅲb期患者[16]。

本研究结果显示，达雷妥尤单抗在难治复发及初治患者中获得了相似的高质量血液学缓解率，分别为63.6％及55.6％。另一方面，28 d内未达到血液学缓解的患者后续亦无法实现缓解，提示作为一种快速起效方案，1个疗程达雷妥尤单抗无效的患者后续难以从中获益，需要尽快调整治疗。而本研究40％的心脏缓解率与既往研究一致。但我们发现，Ⅲb期患者使用达雷妥尤单抗后早期死亡率仍居高不下。我们的既往数据显示，接受硼替佐米治疗的初治Ⅲb期患者3个月及6个月死亡率分别为30.8％及46.2％，仅接受支持治疗的Ⅲb期患者的3个月及6个月死亡率分别为76.9％及84.6％[5]。由此可见，对于Ⅲb期患者，虽然抗浆细胞治疗明显优于支持治疗，但达雷妥尤单抗似乎并未展现出超越硼替佐米的优势。

总体而言，在初治及难治复发心脏AL型淀粉样变患者中，基于达雷妥尤单抗的治疗有较高的安全性，可快速诱导高质量的血液学缓解，进而提升心脏缓解率，但似乎仍难以克服Ⅲb期患者较高的早期死亡率。极高危心脏AL型淀粉样变的治疗策略还需进一步探索。
